# Management of Pulmonary Arterial Hypertension: Current Strategies and Future Prospects

**DOI:** 10.3390/life15030430

**Published:** 2025-03-08

**Authors:** Munish Sharma, Vivek Paudyal, Saifullah Khalid Syed, Rubi Thapa, Nadeem Kassam, Salim Surani

**Affiliations:** 1 Division of Pulmonary, Critical Care and Sleep Medicine, Baylor Scott and White, Temple, TX 76508, USA; munishs1@hotmail.com; 2 Department of General Practice and Emergency Medicine, Karnali Academy of Health Sciences, Chandannath 21200, Jumla, Nepal; paudyalvivek@gmail.com (V.P.); thaparubirubi@gmail.com (R.T.); 3 Department of Medicine, Indiana University School of Medicine, Indianapolis, IN 46202, USA; sksyed@iu.edu; 4 Department of Medicine, Aga Khan University, Nairobi 30270, Kenya; kassamnadeem2@gmail.com; 5 Department of Medicine and Pharmacy, Texas A&M, College Station, TX 77840, USA

**Keywords:** pulmonary arterial hypertension, endothelin receptor antagonists, phosphodiesterase-5 inhibitors, prostacyclin analogs, soluble guanylate cyclase stimulators, activin signaling inhibitors

## Abstract

Primary pulmonary hypertension (PPH), now known as pulmonary arterial hypertension (PAH), has induced significant treatment breakthroughs in the past decade. Treatment has focused on improving patient survival and quality of life, and delaying disease progression. Current therapies are categorized based on targeting different pathways known to contribute to PAH, including endothelin receptor antagonists (ERAs), phosphodiesterase-5 inhibitors (PDE-5 inhibitors), prostacyclin analogs, soluble guanylate cyclase stimulators, and activin signaling inhibitors such as Sotatercept. The latest addition to treatment options is soluble guanylate cyclase stimulators, such as Riociguat, which directly stimulates the nitric oxide pathway, facilitating vasodilation. Looking to the future, advancements in PAH treatment focus on precision medicine involving the sub-stratification of patients through a deep characterization of altered Transforming Growth Factor-β(TGF-β) signaling and molecular therapies. Gene therapy, targeting specific genetic mutations linked to PAH, and cell-based therapies, such as mesenchymal stem cells, are under investigation. Besides prevailing therapies, emerging PH treatments target growth factors and inflammation-modulating pathways, with ongoing trials assessing their long-term benefits and safety. Hence, this review explores current therapies that delay progression and improve survival, as well as future treatments with curative potential.

## 1. Introduction

Pulmonary Hypertension (PH) is a chronic debilitating condition affecting the pulmonary vasculature, characterized by increased pressure of pulmonary circulation. Hemodynamically, it is defined as a mean Pulmonary Artery Pressure (mPAP) at rest of more than or equal to 20 mmHg [1,2].

PH is not a single clinical entity. Instead, it is a cluster of different conditions. Based on the similarity of pathophysiology, hemodynamic parameters, and management options, PH is classified into five groups, as follows [1,2]:

1. Group 1 PH—pulmonary arterial hypertension (PAH);
2. Group 2 PH—PH due to Left Heart Disease;
3. Group 3 PH—PH due to lung diseases and/or hypoxia;
4. Group 4 PH—PH due to pulmonary artery obstructions;
5. Group 5 PH—PH due to multifactorial mechanisms.

The main aim of this article is to revisit current therapeutic approaches to pulmonary hypertension and discuss future treatment options.

Pulmonary arterial hypertension (PAH) is a progressive disease, incurable to date, that ultimately leads to right heart failure and death. PAH is a precapillary PH with hemodynamic features, as shown in Table 1 [2].

A population-based study performed in Canada showed PAH accounting for about 15% of cases of PH, affecting more females (59.7%) as compared to males. The mean age of diagnosis for PAH was 55.4 ± 28 years. In children less than 16 years of age diagnosed with PH, PAH accounted for approximately 65% of cases [3].

Before the advent of PAH-specific therapy in the late 1900s, the median survival of PAH patients was 2.8 years. Only about 68% of patients diagnosed with PAH were able to survive the first year after diagnosis. The survival rates at 3 and 5 years were only 48% and 34%, respectively [4], whereas after about two decades of modern treatment availability, the situation has improved a lot. In 2012, it was found that the median survival of PAH patients was 7 years, with survival rates from diagnosis at 1 year, 3 years, 5 years, and 7 years being 85%, 68%, 57%, and 49%, respectively [5]. This considerable improvement in prognosis was made possible with drugs targeting pulmonary vascular remodeling. Therapies targeting another important prognostic factor, right ventricular failure, are still in the pre-clinical phase, and include metabolic modulation, anti-inflammatory agents, and micro-RNAs-based therapies. A few approved PAH drugs, such as endothelin receptor antagonists (ERAs) and phosphodiesterase-5 inhibitors (PDE-5is), have also shown promising results in terms of improvement in right ventricular failure [6]. A better prognosis of PAH is expected in the future with new drug advances.

Based on etiology, PAH can be further subclassified, as shown in Table 2 [2].

Among the subclassifications, the most common form is Idiopathic PAH, accounting for 39.2% of total cases of PAH, followed by PAH associated with connective tissue disease (15.3%). Other forms, like PAH associated with congenital heart disease (11.3%), PAH associated with portal hypertension (10.4%), PAH associated with anorexigenic (9.5%), PAH associated with HIV (6.2%), and heritable PAH (3.9%), are less common [7].

Histologically, PAH is characterized by diffuse medial hypertrophy and intimal/adventitial thickening. Such lesions suggest the excessive production of vasoconstrictors like endothelin or the loss of vasorelaxants like nitric oxide (NO) and prostacyclin [8]. These findings form the basis for the use of PAH-specific therapies, namely, endothelin receptor antagonists (ERAs), phosphodiesterase 5 inhibitors, guanylate cyclase stimulators, prostacyclin analogs, and prostacyclin receptor agonists.

**Figure 1:**
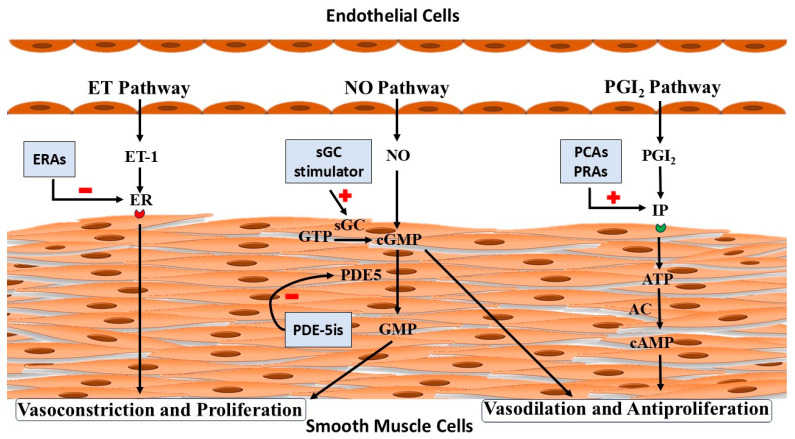
Schematic representation of pathophysiology and pharmacotherapy of pulmonary arterial hypertension.

The pathophysiological overview and site of action of the drugs are shown in Figure 1.

Endothelin (ET) pathway: Endothelin-1 (ET-1) mediates its action via the endothelin receptor (ER). Endothelin receptor antagonists (ERAs) inhibit (−) the action of Endothelin-1 on the endothelin receptor by blocking it.

Nitric oxide (NO) pathway: Nitric oxide acts upon smooth muscles via its second messenger, cyclic Guanosine Monophosphate (cGMP). The soluble Guanylate Cyclase (sGC) stimulator Riociguat stimulates (+) soluble Guanylate Cyclase (sGC) and increases the conversion of Guanosine Triphosphate (GTP) into cyclic Guanosine Monophosphate (cGMP), whereas phosphodiesterase 5 inhibitors (PDE-5is) inhibit (−) the action of phosphodiesterase 5 (PDE5) and prevent the conversion of cGMP into Guanosine Monophosphate (GMP).

Prostacyclin (PGI2) pathway: Prostacyclin mediates its action via the prostacyclin receptor (IP). Prostacyclin analogues (PCAs) and prostacyclin receptor agonists (PRAs) act upon the prostacyclin receptor and stimulate the production of cyclic Adenosine Monophosphate (cAMP). AC: Adenylyl cyclase. ATP: Adenosine Triphosphate.

## 2. Endothelin Receptor Antagonists (ERAs)

Endothelin is a potent vasoconstrictor with four isoforms. Endothelin-1 (ET-1) is the most common isoform produced by endothelial cells, which acts upon vascular smooth muscle cells and endothelial cells in an autocrine or paracrine fashion [9,10]. It leads to vascular remodeling, resulting in medial hypertrophy and plexiform lesions in patients with PAH [8]. The actions of endothelin are mediated via endothelin receptors (ER). ET_A_ is the isoform of the endothelin receptor most abundantly present in vascular smooth muscles, and it contributes to vasoconstriction and cellular proliferation. In contrast, ET_B_ is primarily present in endothelial cells, and contributes to endothelin clearance and vasodilation [9,10].

ERAs act by blocking the actions of endothelin at endothelin receptors, mainly ET_A_. Bosentan and Macitentan are dual endothelin receptor antagonists with blocking effects at both ET_A_ and ET_B,_ whereas Ambrisentan only antagonizes ET_A_ [11,12,13].

Bosentan is the pioneer drug in the group, approved by the FDA in 2001 based on the results of the BREATHE-1 trial in patients with Idiopathic PAH and PAH associated with connective tissue diseases. It showed improvement in the 6 min walking test (6 MWT), World Health Organization (WHO) functional class, and time to clinical worsening. The efficacy of Bosentan was hsown to be similar in both the dosage forms used in the trial (125 mg twice daily or 250 mg twice daily). In contrast, the most common side effect, i.e., abnormal hepatic function, was dose-dependent.

About 10% of patients suffered from an increase in hepatic enzymes, whereas this was 7% in patients taking Bosentan 250 mg twice daily [14]. Thus, the recommended maximum dosage of Bosentan for the treatment of PAH is 125 mg twice daily [1].

Ambrisentan, the second drug in the group, was approved by the FDA in 2007 in patients with Idiopathic PAH, Heritable PAH, and PAH associated with connective tissue diseases. In contrast to Bosentan, it has once-daily dosing [15]. ARIES-1 and ARIES-2, concurrent randomized–controlled trials, showed improvement in the 6 min walk test (6 MWT) after 12 weeks of treatment with different dosages of Ambrisentan, the maximum dose being 10 mg once daily. The average change from baseline in 6 min walk distance was 40 m (95% Confidence Interval, 33 to 48 m) in week 12 and 39 m (95% CI, 29 to 49 m) at week 48, thus suggesting a sustained improvement in primary endpoint caused by the continued use of Ambrisentan. The most worrying adverse effects of Bosentan, i.e., raised liver enzymes, were not seen with Ambrisentan. Peripheral edema, headache, and nasal congestion were observed in patients treated with Ambrisentan due to systemic vasodilation. Nasal congestion was the only side effect found to have a dose-response relationship. The ARIES study showed that 5 mg and 10 mg once-daily dosages showed favorable efficacy on safety profile. Improvements in exercise capacity (6 MWT) showed a positive dose–response trend, which supports the clinical decision to increase the dose from the initial 5 mg once daily to 10 mg once daily if the drug is well tolerated [16].

Similarly, Macitentan, the latest addition to the group in 2013, is an oral formulation with once-daily dosing that antagonizes both the receptor isoforms. The SERAPHIN trial showed that long-term treatment with Macitentan reduces morbidity and mortality among patients with PAH. Unlike previous short-term trials of 12–16 weeks considering improvements in exercise capacity as the primary endpoint, the SERAPHIN trial used the time from the initiation of treatment to the first event related to PAH (worsening of PAH, initiation of treatment with intravenous or subcutaneous prostanoids, lung transplantation, or atrial septostomy) or death from any cause up to the end of treatment as their primary endpoint. A total of 742 patients were enrolled in this multicenter trial, suffering from Idiopathic PAH, Heritable PAH, drug-/toxin-induced PAH, PAH associated with connective tissue diseases, PAH with repaired congenital left to right shunts, and PAH with HIV infection. Over the median period of 115 weeks, about half of patients receiving a placebo had events related to PAH or death due to any cause. The percentage was lesser with patients receiving Macitentan (38.0% and 31.4% in patients receiving 3 mg and 10 mg, respectively), demonstrating a significant reduction in morbidity compared to placebo. The trial also showed improvements in exercise capacity and WHO Functional class, as in previous trials. The most worrying adverse effects of hepatic transaminitis did not appear to be significant in this trial, as both placebo and drug groups showed similar increments in the level of liver enzymes. Systemic vasodilation causing headache and nasopharyngitis were of concern. Anemia is one of the more significant adverse effects of Macitentan, and 13.2% and 8.8% of patients treated with 10 mg and 3 mg of Macitentan suffered from anemia, in contrast to 3.2% of those receiving placebo. Unfortunately, the trial was not sufficiently powered to show any mortality benefits [17].

A meta-analysis on the safety of ERAs in the treatment of PAH studied the adverse effects of three ERAs. Table 3 shows the relative risks (RRs) of three different ERAs concerning their most common adverse effects.

It demonstrated that Bosentan 125 mg twice daily had the highest risk of abnormal liver function, Ambrisentan 10 mg once daily had the highest risk of peripheral edema, and Macitentan 10 mg had the highest risk of anemia [18].

ERAs can be considered as initial monotherapy in patients having PAH with cardiac comorbidities, while in patients with PAH without cardiac comorbidities, ERAs are part of initial combination therapy [1]. Real-world challenges related to using endothelin receptor antagonists (ERAs) in pulmonary hypertension include long-term adherence issues due to side effects such as liver toxicity, peripheral edema, and anemia, which often lead to treatment discontinuation. Drug interactions are another concern, as ERAs like Bosentan can induce cytochrome P450 enzymes, affecting the metabolism of anticoagulants and other cardiovascular drugs. Additionally, treatment resistance remains a significant hurdle, with some patients exhibiting suboptimal responses despite optimal dosing, possibly due to variations in endothelin receptor expression or downstream signaling pathways [1,19].

## 3. Nitric Oxide-Cyclic Guanosine Monophosphate (cGMP) Stimulators

The nitric oxide-cyclic guanosine monophosphate pathway is fundamental for pulmonary vascular regulation, the mediation of vasodilation, the reduction in vascular remodeling, and the inhibition of platelet aggregation. The dysregulation of this pathway can lead to increased PVR. Hence, this pathway is a prime therapeutic target.

Nitric oxide (NO) is synthesized from L-Arginine with the help of the enzyme NO-Synthase (NOS). There are three isoforms of NOS, namely, neuronal NOS (nNOS), inducible NOS (iNOS), and endothelial NOS (eNOS). Endothelial NOS is expressed by most endothelial cells and is responsible for producing NO, which has vasodilatory, anti-thrombotic (anti-platelets aggregatory), and anti-proliferative effects. NO acts via its second messenger, cGMP, produced by the action of soluble Guanylate Cyclase (sGC) upon GTP. This cGMP is reduced to GMP with no residual functions of cGMP by the action of the enzyme named phosphodiesterase (PDE), as shown in Figure 1 [20]. PDE-5 is the isoform of PDE most highly expressed in pulmonary artery smooth muscle cells. It is responsible for the hydrolysis of about 80% of cGMP produced in circulation. PDE-5 is overexpressed in pulmonary circulation smooth muscle cells in patients with PAH, and forms the basis for treating PAH with PDE-5 inhibitors (PDE-5is) [21].

Two major classes of medications modulate the NO-cGMP pathway; phosphodiesterase type 5 (PDE-5) inhibitors, which prevent cGMP degradation, and soluble guanylate cyclase (sGC) stimulators, which enhance cGMP synthesis independently of NO availability.

### 3.1. Phosphodiesterase-5 Inhibitors (PDE-5i)

PDE-5i was initially approved for use in the context of erectile dysfunction. Sildenafil and Tadalafil are the most commonly used PDE-5is that were later used for PAH based on different clinical trials.

Sildenafil, a PDE-5 inhibitor, was the second oral drug to be studied for use in PAH therapy after Bosentan. SUPER-1, a 12-week, multicentric, double-blinded, placebo-controlled trial, was designed to study the efficacy and safety of using Sildenafil for PAH therapy, which included 278 patients with Idiopathic PAH, PAH associated with connective tissue diseases and PAH with repaired congenital systemic to pulmonary shunts. All patients were randomized into four groups, i.e., placebo and Sildenafil at a dosage of 20 mg three times daily, 40 mg three times daily, or 80 mg three times daily.

Improvement in 6 min walk distance (6 MWD) was considered the primary endpoint. Sildenafil demonstrated improvement in 6 MWT irrespective of the dosage used. The mean placebo-corrected improvements were 45 m, 46 m, and 50 m with 20 mg, 40 mg, and 80 mg of Sildenafil, respectively. Sildenafil showed statistically significant improvements in hemodynamic parameters, i.e., a decrease in mPAP and pulmonary vascular resistance (PVR). However, there was no delay in time to clinical worsening with Sildenafil as compared to placebo, while an improvement in WHO functional class was seen [17]. The extension period in the SUPER-1 trial showed the mean change in 6 MWD to be 51 m after 12 months of monotherapy, suggesting its sustained effect. The SUPER-2 trial, an open-label uncontrolled extension of SUPER-1, demonstrated that 46% of patients had increased 6 MWD after 3 years of treatment, and 29% of patients had an improved WHO-FC, while 31% were able to remain in the same WHO-FC without further clinical deterioration with monotherapy. Patients with baseline 6 MWD < 325 m in the SUPER-1 trial and those who showed no improvement in 6 MWD even after 12 weeks of treatment with Sildenafil had poor survival. Thus, close monitoring and early aggressive treatment options could benefit such cases [22].

Mostly mild to moderate side effects related to PDE-5is were observed during the trial, such as headache, dyspepsia, diarrhea, blurred vision, etc. Left ventricular dysfunction and postural hypotension were serious adverse effects observed in one patient during the first 12 weeks of the study period. Other perceived serious adverse effects included seizures, drug hypersensitivity, angioedema, and subcapsular cataracts. Though the drugs in different dosages were well tolerated, no dose–response relationship was evident regarding efficacy outcomes, likely due to the complete inhibition of PDE-5 with the lowest dose. Thus, 20 mg three times daily is the only approved dose of Sildenafil based on the statistical benefit seen in the 12-week trial [22,23].

Like Sildenafil, Tadalafil’s safety and efficacy were studied with different dosage forms (2.5 mg, 10 mg, 20 mg, and 40 mg once daily). The PHIRST trial included 405 patients with Idiopathic PAH, Heritable PAH, drug- and toxin-induced PAH, PAH associated with connective tissue disease, HIV, and congenital left to right shunt. Tadalafil manifested improvements in 6 min walk distance (6 MWD) with different dosage forms (except 2.5 mg once daily) compared to placebo. Although the improvements were clinically significant, only 40 mg OD achieved a pre-specified statistically significant value. Unlike Sildenafil, Tadalafil demonstrated a delay in time to clinical worsening, whereas no improvement in the WHO functional class was seen. Improvements in hemodynamic parameters like mPAP and PVR were observed, as with Sildenafil. Subgroup analysis in patients taking Tadalafil 40 mg revealed that the placebo-adjusted change in 6 MWD was 44 m in treatment-naïve patients. In contrast, it was only 23 m in patients with background Bosentan therapy. Though the exact mechanism is unclear, it is thought that the pharmacokinetic interaction between Bosentan and Tadalafil, mediated via Cytochrome P450, is responsible for the decreased efficacy of Tadalafil in combination therapy [24,25].

The long-term extension (PHIRST-2) revealed sustained improvement in the exercise capacity of patients taking Tadalafil regularly [26]. The most common side effects observed during the 16-week trial were headache, myalgia, and flushing with mild to moderate intensity. Serious side effects like nausea, vomiting, retinal artery occlusion, dyspnea, priapism, esophageal varices hemorrhage, hypotension, gastritis, menorrhagia, histiocytosis hematophagic syndrome, and drug hypersensitivity were rare. The prevalence of headaches was lower in long-term extensions than in the initial 16-week trial, suggesting that the side effects waned with time [24,26]. As the improvement in exercise capacity was dose-dependent, whereas the side effects were similar in all dosage forms, 40 mg once daily was approved as the dosage form of Tadalafil for PAH therapy [24,26].

A meta-analysis of PAH patients treated with PDE-5is has shown that the treatment with PDE-5is will likely improve WHO-FC and exercise capacity in patients measured in terms of 6 MWD. About 22% of cases are less likely to die within 14 weeks of treatment with PDE-5i as compared to placebo. It also showed that the treatment’s most common adverse effects are headache, GI upset, flushing, muscle aches, and joint pains [27]. Like ERAs, PDE-5is are also considered a first-line monotherapy for patients with PAH with cardiac comorbidities. They are considered for use in combination therapy with ERAs in cases of low- to intermediate-risk patients, and in combinations with ERAs and prostacyclin analogs in high-risk PAH cases [1]. Some patients fail to respond to PDE5 inhibitors (PDE5i) due to advanced pulmonary vascular remodeling, right ventricular dysfunction, or suboptimal dosing. Pharmacokinetic issues, such as poor absorption or drug interactions, and cGMP dysregulation from endothelial dysfunction or increased PDE5 expression, can also contribute to PDE5 inhibitor failure. PDE5is are less effective in the context of non-PAH causes of pulmonary hypertension (e.g., group 2 or group 3 PH). Some patients develop tolerance or tachyphylaxis, reducing long-term efficacy. Potential solutions include combination therapy (e.g., PDE5i + endothelin receptor antagonists) and switching to alternative vasodilators like riociguat.

### 3.2. Soluble Guanylate Cyclase (sGC) Stimulator

Riociguat is the sole drug in the group that stimulates the production of cGMP and promotes the action of nitric oxide (NO). Its phase II trial demonstrated promising efficacy and safety for use in both Group 1 and Group 4 PH. It became one of the drugs to be approved for treatment in two different groups of PH [28]. In the PATENT trial, 443 patients were enrolled with Idiopathic PAH, Heritable PAH, drug- or toxin-induced PAH, PAH associated with connective tissue disease, congenital heart disease, and portal hypertension with cirrhosis. Here, 50% of the participants were treatment-naïve, whereas 44% received ERA and 6% received Iloprost as background therapy. Riociguat was used in different individualized dosage forms, the maximum dose being 2.5 mg three times daily. It showed an improvement in 6 min walk distance (6 MWD) at 12 weeks in both subgroups of patients, treatment-naïve and those under background therapy [29]. The 6 MWD was improved over long-term extension by 51 m at 1 year and 47 m at 2 years, suggesting its sustained effect [30,31]. Though Riociguat is equally effective when used in treatment-naïve patients and patients with background therapy, it is not recommended to combine Rioiciguat with PDE-5is due to the increased risk of hypotension [32].

Improvements in hemodynamic parameters (mPAP and PVR), NTproBNP levels, WHO-FC, and time to clinical worsening were also seen in the 12-week trial of Riociguat [29]. There was an improvement in WHO-FC in about 33% of patients, and 61% of patients’ WHO-FCs were maintained at the end of 1 year of treatment with the drug [30]. Syncope, worsening PH, chest pain, and right ventricular failure were common adverse effects seen. The worrisome adverse effects were hemoptysis and pulmonary hemorrhage. In the PATENT-2 study, 10 patients (3%) suffered from hemoptysis and pulmonary hemorrhage, among which, in 7 (2%) cases, it was resolved without drug withdrawal or dose reduction, whereas 1 patient (0.3%) died of it, 1 patient (0.3%) underwent drug withdrawal and death later, and in 1 patient (0.3%) the issue was not resolved within the study period. Though the exposure-adjusted rate per 100 patient years for pulmonary hemorrhage was 1.8 in PATENT-2 as compared to 4.3 in PATENT-1, the potential risk of bleeding with the drug is a concern [30].

The survival rate for 1 year was estimated to be 97%, and the incidences of clinical worsening and hospitalization due to PAH were relatively low (21% and 10%, respectively). These results are in favor of the long-term efficacy of Riociguat in patients with PAH [30].

Table 4 shows a comparison between PDE-5 inhibitors and sGC stimulators.

### 3.3. Prostacyclin Analogues and Receptor Agonists

Prostacyclin (PGI2) and its analogues are potent vasodilators with anti-proliferative, anti-thrombotic, anti-inflammatory, and anti-platelet aggregatory effects. They mediate their actions via a prostacyclin (IP) receptor-stimulating G protein-coupled increase in cAMP [32]. In PAH patients, Prostacyclin Synthase (PGI2-S), responsible for the synthesis of PGI2, is decreased in the pulmonary circulation, which could be the reason for thrombosis and inflammatory changes in vessels [33]. Currently, three prostacyclin analogs are used for the treatment of PAH patients, viz. Epoprostenol, Treprostinil and Iloprost.

Epoprostenol is a synthetic analog of prostacyclin with a very short half-life of <3–5 min (like that of endogenous prostacyclin), requiring intravenous (IV) administration for its action. As it irritates the vein, the peripheral IV route is not preferred; rather, administration via a continuous IV infusion through a central venous catheter is preferred [34]. It was the first drug to be used for the treatment of PAH, and is a significant therapy. The first trial with Epoprostenol was conducted in 1990, and it was randomized for the first 8 weeks, followed by an unrandomized extension of 18 months. It showed improvements in hemodynamic parameters like PVR and mPAP, which were sustained for 18 months but required dose adjustments [35]. Later, 81 patients with PAH in WHO-FC III/IV were enrolled for a 12-week trial, where Epoprostenol combined with conventional therapy (anticoagulants, vasodilators, diuretics, digoxin) versus traditional therapy alone was studied. Patients in the treatment group showed statistically significant improvements in exercise capacity. The mean 6 MWD improved by 32 m in the treatment group versus a decrease of 15 m in a conventional therapy group. Further, 40% of patients showed improvements in their WHO-FC, and 48% maintained their WHO-FC without deterioration. Hemodynamic parameters like mPAP, PVR, and Cardiac Index (CI) were significantly improved. Eight patients died during the 12-week trial, and all deaths were from the conventional therapy group. The 6 MWD at baseline was significantly lower in the dead patients when compared to that of survivors, thus indicating 6 MWD as an independent predictor of survival in PAH patients [36]. The survival benefit of Epoprostenol found in short-term studies was not limited. Survival with Epoprostenol therapy at 1, 2, and 3 years was 87.8%, 76.3%, and 62.8%, respectively, and was significantly better than the expected survival of 58.9%, 46.3%, and 35.4%, respectively, based on historical data [37]. Patients with a history of right heart failure, the absence of significant hemodynamic improvement after 3 months of Epoprostenol therapy, and persistence of WHO-FC after 3 months of treatment tended to show poor survival. They could thus be suitable candidates to be considered for lung transplantation [38]. The common side effects of Epoprostenol use were jaw pain, diarrhea, flushing, headache, nausea, vomiting, etc., which are mainly related to its vasodilatory effects. The serious side effects to be considered were catheter-related sepsis, catheter-related thrombotic events, infection at the catheter site, and bleeding or pain at the catheter site. Interruption in continuous IV Epoprostenol has been shown to increase symptoms in patients, leading to clinical worsening [36]. The main limitation of Epoprostenol therapy is the difficulty in drug handling and its delivery process.

Unlike Epoprostenol, with molecular instability and a short half-life, Treprostinil is a stable compound with a half-life of about 4.5 h. It is available in four forms related to administration, i.e., subcutaneous (SC), intravenous (IV), inhalation, and oral. Subcutaneous Treprostinil was approved for the treatment of PAH patients with WHO-FC II-IV in 2002 [34]. A 12-week double-blind, placebo-controlled, multicentric trial was performed with 470 patients suffering from Idiopathic PAH and PAH associated with connective tissue disease and congenital heart disease between November 1998 and October 1999. The initiation dose for the continuous subcutaneous infusion of Treprostinil was 1.25 ng/kg/min, and the maximum allowable dose in 12 weeks was 22.5 ng/kg/min. The treatment group showed only a modest improvement in 6 MWD (the difference in median distance walked was 16 m), but this was statistically significant. Interestingly, the improvement in exercise capacity measured by 6 MWD was observed to be significantly improved in those with a poor capacity at baseline. The inclusion of relatively stable patients in this trial compared to those on Epoprostenol might be the reason for the modest improvement in exercise capacity. Moreover, the most common adverse effect, i.e., infusion site pain, prevented the adequate increase in the dosage of Treprostinil in some patients [39]. This trial showed improvements in multiple other hemodynamic parameters and clinical signs and symptoms of patients. Improvements in patient outcomes were dose-dependent, with the maximum improvement seen in patients receiving dosages of >13.8 ng/kg/min. Though this trial did not demonstrate a mortality benefit, it yielded significant improvement, and suggests this treatment could act as an alternative to the tiresome administration of Epoprostenol, with multiple catheter-related complications. The most common adverse effect of SC Treprostinil was infusion site pain; 85% of patients had this side effect, and 8% of cases discontinued the treatment. It did not appear to be dose related, but instead correlated with the rate of dose increment. Other significant side effects were irritation, bleeding, and bruising at the infusion site, and side effects related to the vasodilatory effects of the drug included jaw pain, flushing, and edema. Unlike Epoprostenol therapy, an interruption in drug delivery due to infusion system malfunction did not lead to any serious adverse effects in patients with SC Treprostinil. Though three episodes of GI bleeding were noted, they were uneventful and were not considered to be due to Treprostinil. Instead, they were thought to be due to other conventional therapies [39]. Long-term therapy with the drug is related to continuous improvements in exercise capacity and clinical symptoms in patients with PAH. Event-free survival rates, i.e., survival without events like hospitalization for clinical worsening, transition to IV Epoprostenol, and need for combination therapy or atrial septostomy, were 83.2% and 69% at 1 year and 3 years, respectively, which appear to be similar to those of IV Epoprostenol [40].

Among the different routes of administration of Treprostinil, the FDA has approved SC, IV and inhalation routes for the treatment of PAH. A 12-week open-label, multicentric study including 16 patients with Idiopathic PAH, PAH associated with connective tissue disease, and PAH associated with congenital heart disease in WHO-FC III and IV demonstrated improvements in exercise capacity, dyspnea, and hemodynamic parameters with IV Treprostinil. The mean increment in 6 MWD was 82 m, while it was only 16 m in the treatment group with SC Treprostinil. There was no deterioration in WHO-FC during the study, and no patients were in WHO-FC IV by the end of the treatment period. The improvement in dyspnea became more significant with more rapid dose escalations, and higher dosages were given to the patients than in the previous trial on the SC route [41]. The IV administration of Treprostinil is advantageous over IV Epoprostenol because of its longer half-life and stability at room temperature. The chance of a life-threatening crisis due to an inadvertent infusion interruption is less compared to IV Epoprostenol because of its longer half-life. Besides this, the storage and preparation of infusions are less laborious, and can enhance patient convenience. The transition from Epoprostenol to Treprostinil is also found to be safe and effective [41,42]. The most worrisome adverse effects of IV Treprostinil were extremity pain and jaw pain, reported by 69% of participants. Other side effects, such as nausea, headache, flushing, and diarrhea, were minor. The main issue with SC Treprostinil, i.e., infusion site pain, is not seen with the IV route, thus improving patient compliance [41]. The continuous intravenous (IV) infusion of prostacyclin analogs, such as epoprostenol or Tresprotinil, presents significant practical challenges, including catheter-related infections, thrombosis, and pump malfunctions, which can lead to life-threatening interruptions in therapy. In contrast, subcutaneous prostacyclin therapy, like treprostinil, eliminates the need for a central venous catheter, reducing infection risks, but it is often limited by severe infusion site pain, which can be a major barrier to adherence. While both delivery methods offer life-prolonging benefits in pulmonary arterial hypertension, the choice between them requires balancing the risks of catheter-related complications with the tolerability of subcutaneous administration [37,38,43].

Inhaled Treprostinil was also found to be an effective adjunct therapy in patients who remained symptomatic despite treatment with ERAs or PDE-5is. A 12-week trial showed a placebo-corrected median change from baseline in 6 MWD of 20 m [44]. Along with inhalation via nebulization, a dry powder inhalation (DPI) form has been FDA-approved for use in PAH treatment. Transition from nebulization to the DPI form was demonstrated to be safe and convenient in the BREEZE trial [45]. Compared to the SC and IV administration of prostacyclin, a more straightforward route such as inhalation can aid in initiating prostacyclin earlier in the treatment plan. Inhaled Treprostinil has also been approved by the FDA for the treatment of Group 3 PH [46,47].

Iloprost is an FDA-approved inhalation therapy for PAH. A 12-week, multicentric, placebo-controlled trial with patients suffering from severe PAH and chronic thromboembolic PH (CTEPH) showed significant improvements in exercise capacity and functional classification. This study used a combined primary endpoint of at least 10% improvement in 6 MWD and improvement in WHO-FC (then termed NYHA FC). Here, 16.8% of participants met the primary endpoint, whereas in about 40% of patients, the improvement in 6 MWD was more than 10%. The mean increase in 6 MWD was 36.4 m, but when only primary pulmonary hypertension was taken into account, it was 58.8 m. There was no significant improvement in hemodynamic parameters, but no further deterioration was seen. The mean dosage for inhalation was 0.37 ng/kg/min, which is comparatively less, and it is assumed that due to the direct action of the inhalation route over pulmonary circulation, its potency is higher as compared to other forms of administration [48]. The disadvantage of the inhalation route is the requirement of repeated inhalation of the drug about six to nine times per day via nebulization. Aside from the side effects related to prostacyclin, syncopal attack was considered to be a severe problem in patients treated with Iloprost. It usually occurred in patients within 2–9 h of last therapy, and could be due to the loss of the pharmacological effect of Iloprost [48]. Iloprost has shown efficacy and safety not only as an add-on to conventional treatment, but also as efficacious and safe when used in combination with Bosentan [48,49].

Selexipag, an oral prostacyclin (IP) receptor agonist, is chemically different from prostacyclins, yet is considered part of the group because of its similar pharmacological action via the prostacyclin receptor. In PAH patients undergoing stable treatment with ERA and/or PDE-5i, the addition of Selexipag showed a statistically significant improvement of 30.3% in PVR after 17 weeks [50]. The GRIPHON trial, an event-driven, phase III RCT, studied Selexipag in patients with Idiopathic PAH, Heritable PAH, drug- and toxin-induced PAH, and PAH associated with connective tissue disease, HIV, and congenital heart disease. The primary endpoint for the study was a composite of death from any cause or complication related to PAH up to the end of treatment; 41.6% of patients in the placebo group and 27% in the treatment group met the primary endpoint. Disease progression and hospitalization accounted for 81.9% of events. No significant difference in mortality was seen between the two groups. The dosage of Selexipag was individualized and variable in the study. The initial dose was 200 micrograms twice daily, increasing until adverse effects were observed, and the maximum tolerated dose was calculated. The maximum dose in this trial was 1600 micrograms twice daily. Efficacy was similar among patients receiving low, medium, and high doses. Similarly, the drug was found to have a similar effect whether the patient was undergoing stable background therapy with ERA, PDE-5i, or both, or no treatment at all. Adverse effects were related to the vasodilatory effects of the drug. Headache, diarrhea, and nausea were significant side effects that even led to discontinuation of the therapy [51].

The 2022 European Society of Cardiology (ESC)/European Respiratory Society (ERS) guidelines for diagnosing and treating pulmonary hypertension have recommended using a combination therapy of ERA and PDE-5i for patients with low or intermediate risk and without cardiac complications [1].

Combination with Bosentan has been limited due to drug interactions, as discussed earlier, whereas the combination of Tadalafil with Ambrisentan and Macitentan showed promising results. In an event-driven, randomized–controlled trial, it was observed that in a treatment-naïve patient, initial combination therapy with Ambrisentan and Tadalafil resulted in a significantly lower risk (50% lower risk) of clinical failure events with Ambrisentan or Tadalafil monotherapy. Thus, targeting multiple pathways with early combination therapy could benefit patients with PAH [52].

### 3.4. Fixed-Dose Combination Drug (Macitentan/Tadalafil)

A DUE study, a multicentric, double-blind, adaptive phase III trial, studied the efficacy and safety of combination therapy in the form of a Fixed Dose Combination over 16 weeks. Patients suffering from PAH in WHO-FC II and III were included in the trial. They were randomized into three groups, receiving Macitentan 10 mg/Tadalafil 40 mg Fixed-Dose Combination (M/T FDC), Macitentan 10 mg monotherapy, or Tadalafil 40 mg monotherapy. There was a significant improvement in PVR in subjects treated with M/T FDC as compared to monotherapy. Changes in PVR from baseline to week 16 are shown in Table 5 [53].

There was an improvement in exercise capacity measured in terms of 6 MWD in patients from all groups, but no statistically significant differences were observed when compared between the groups. The improvements in symptoms and WHO-FC were also not significantly different among the groups [53]. In a preclinical study, unlike monotherapy, the combination of Macitentan and Tadalafil showed improvements in pulmonary remodeling [6].

Headache and peripheral edema were the most common treatment-emergent adverse effects. Anemia, hypotension, and edema were more common in the M/T FDC group than others. No new or unexpected adverse effects were observed during the trial, and the drug was well tolerated. Owing to the reduction in pill burden with the benefit of combination therapy, the study supported the use of single-tablet combination therapy for initial management in patients with PAH [53].

The upfront combination therapy with Ambrisentan and Tadalafil has demonstrated the strongest efficacy in pulmonary arterial hypertension (PAH), significantly reducing morbidity and improving hemodynamics, as shown in the AMBITION trial [52]. Bosentan and Sildenafil are less favored due to drug interactions. Bosentan induces CYP3A4, reducing Sildenafil levels and potentially diminishing its efficacy. The fixed-dose Macitentan/Tadalafil combination simplifies adherence and ensures optimal dosing, but requires real-world validation against sequential therapy, where patients are often escalated based on disease progression.

Table 6 summarizes the different treatment options currently available, with comparisons of their cost.

New therapeutic options are needed in the context of pulmonary arterial hypertension (PAH) because existing treatments primarily target symptoms and are not curative. Current therapies, such as endothelin receptor antagonists, phosphodiesterase inhibitors, and prostacyclin analogs, help manage vasodilation and pulmonary pressure, but often do not prevent disease progression or address underlying pathophysiology. Additionally, these treatments can have significant side effects, require complex dosing regimens, and may not be effective for all patients. There is also a need for therapies that address different stages of the disease and target novel mechanisms for better long-term outcomes.

## 4. Activin Signaling Inhibitor

Activin and bone morphogenetic protein (BMP) are Transforming Growth Factor β (TGF-β) family members [61]. Activin A is thought to increase proliferation and induce the gene expression of Endothelin-1 and Plasminogen activator inhibitor-1 in pulmonary artery smooth muscle cells, ultimately contributing to vascular remodeling [62]. On the other hand, BMP 2 is antiproliferative, and can inhibit smooth muscle cell proliferation without stimulating extracellular matrix synthesis [63]. In normal individuals, the expression of BMP Receptor (BMPR2) is prominent on vascular endothelium, whereas in PAH cases, especially those with heterozygous BMPR2 mutations, its expression is markedly reduced in peripheral lung tissue, and the expression of Activin A is found to be increased [64,65]. Activin A mediates its action by binding at its Activin Receptor Type II A (ActRIIA) [61,65].

**Figure 2:**
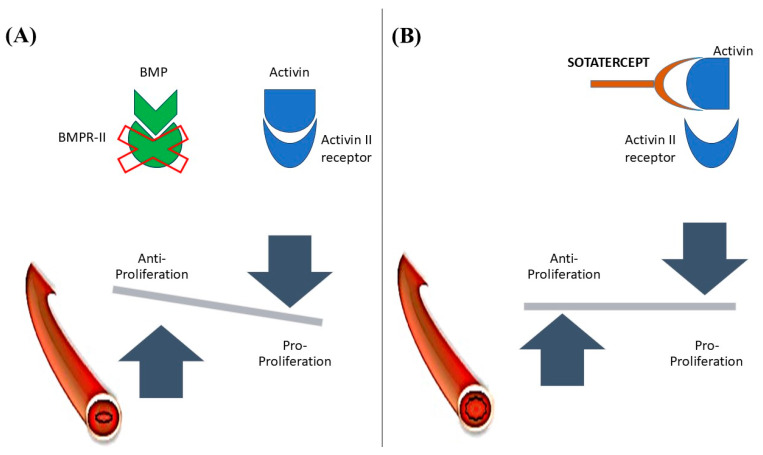
(**A**) Unbalanced action of Activin due to bone morphogenetic protein receptor II (BMPR-II) mutation leads to increased proliferation. (**B**) Sotatercept binding with Activin counteracts the proliferative effect of Activin. BMP: bone morphogenetic protein.

A pathophysiologic overview of the action of Sotatercept is shown in Figure 2.

Sotatercept, a decoy Activin receptor, is a fusion protein (ActRIIA-Fc) composed of the extracellular domain of human Activin Receptor Type II A fused with the Fc portion of IgG1 [65]. It prevents the binding of Activin A to its receptor, and prevents vascular remodeling in the case of PAH. It is an FDA-approved drug that has shown promising results in phase II and III trials. Those trials included patients with PAH (Idiopathic PAH, Heritable PAH, drug-/toxin-induced PAH, PAH associated with connective tissue disease and congenital heart disease) in WHO-FC II and III who were undergoing stable background therapy for at least 3 months with PAH-specific drugs [66,67]. Sotatercept was given subcutaneously every 3 weeks, and its efficacy and safety were measured. It showed significant improvement in PVR and WHO functional classes. The difference between the treatment group and placebo in terms of change in 6 MWD from baseline at 24 weeks was 40.8 m [66,67,68]. The risk of death or nonfatal clinical worsening events, assessed up to the end of the trial, was 84% lower with sotatercept than with placebo [67]. Thrombocytopenia was the most common adverse effect of interest during the trial, yet no thrombocytopenia-induced bleeding occurred, nor was platelet transfusion required. However, thrombocytopenia resulted in the discontinuation of Sotatercept in one patient, and another withdrew their consent. Erythrocytosis was another effect of concern; three patients had to withdraw from the trial as their hemoglobin level rose above 18 mg/dl, and they underwent phlebotomy [66]. Thus, Sotatercept had a favorable benefit-to-risk ratio when combined with stable background PAH-specific therapy. It could be a helpful add-on therapy for patients refractory to PAH treatment.

The discussion of current treatment options available for PAH is summarized in Table 7.

A summary of management principles and treatment considerations in different PAH subclasses is shown in Table 8.

## 5. Treatment Algorithm

As discussed, there are multiple pharmacological options for the treatment of PAH. The decision regarding the treatment plan is based on pretreatment assessment for baseline risk. Three strata models segregating the patients into low-, intermediate- and high-risk groups are used in the initial treatment plan. This was suggested by the 2015 ESC/ERS Guidelines for the diagnosis and treatment of PH. It represents a comprehensive model, and includes different parameters like clinical features of right heart failure, progression of symptoms, syncope, 6 MWD, WHO-FC, biomarkers (BNP/NTproBNP), different parameters of echocardiography, cardiac MRI, cardiopulmonary exercise testing (CPET) and hemodynamic parameters.

A few abbreviated approaches of the three-strata risk stratification tool have been validated using the Swedish Pulmonary Arterial Hypertension Registry (SPAHR), the Comparative, Prospective Registry of Newly Initiated Therapies for PH (COMPERA), and the French PH Registry (FPHR) [1,75,76,77]. The United States Registry has developed other risk-stratification tools to evaluate early and long-term PAH disease management (US REVEAL), including the REVEAL2.0 risk score calculator and REVEAL Lite 2 [1,78,79]. The objective of all these stratification models is to determine whether the patient is at high risk, which is essential for making decisions regarding treatment initiation with prostacyclin or other medications.

**Figure 3:**
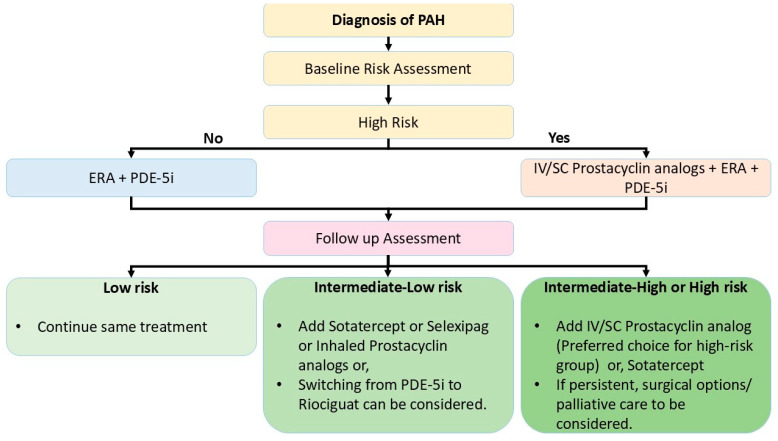
Treatment algorithm for PAH patients. ERA: endothelin receptor antagonist. IV: intravenous. PDE-5i: phosphodiesterase 5 inhibitor. SC: subcutaneous.

A four-strata model is proposed for risk assessment for follow-up cases under treatment. Its simplified version only requires WHO-FC, 6 MWD, and BNP/NTproBNP levels for calculation. The treatment algorithm using risk assessment strategies is shown in Figure 3 [1,80].

## 6. Surgical Strategies in Severe Pulmonary Arterial Hypertension (PAH)

### 6.1. Risk Assessment and Decision-Making for Treatment

Pulmonary arterial hypertension (PAH) represents a challenging condition requiring a multidisciplinary approach. Severe PAH (WHO functional class III/IV) is associated with poor survival despite advanced medical therapies. Surgical options become crucial in these patients, aiming to palliate symptoms, improve hemodynamics, and enhance quality of life. Decision-making for surgical intervention involves complex considerations, including risk assessment, timing of intervention, and patient-specific factors [1,81].

Effective risk stratification is critical for identifying surgical candidates in severe PAH. Key components include the following [1]:

Hemodynamic parameters. Right atrial pressure (RAP), cardiac index (CI), and pulmonary vascular resistance (PVR) are pivotal hemodynamic predictors. RAP > 15 mmHg and CI < 2 L/min/m^2^ indicate high-risk patients unsuitable for extensive surgeries;

Biomarkers. Elevated NT-proBNP and troponin levels correlate with right ventricular dysfunction and adverse outcomes;

Functional capacity. The 6 min walk test (6 MWT) and cardiopulmonary exercise testing (CPET) are standard assessments used to evaluate functional reserve and surgical candidacy.

#### Surgical Strategies

A. Right to Left Shunting

Right to left shunting increases systemic blood flow due to increased left ventricle preload from right ventricle decompression. This procedure involves atrial septostomy and the Potts shunt [81].

I. Atrial septostomy is a palliative intervention used in patients with severe pulmonary arterial hypertension (PAH) who have right ventricular (RV) failure refractory to medical therapy. It involves creating a right-to-left shunt at the atrial level to decompress the right heart, reduce right atrial pressure, and increase systemic cardiac output. This results in improved oxygen delivery despite systemic desaturation. Hemodynamic stability during the procedure is critical for success. Risks include systemic desaturation, paradoxical embolism, and worsening left ventricular filling in the setting of reduced LV compliance [81,82,83,84].
II. Potts shunt, a surgical alternative, is considered for pediatric or selected adult patients with supra-systemic PAH and right ventricular failure. The procedure involves creating a left pulmonary artery-to-descending aorta anastomosis and establishing a right-to-left shunt that offloads the RV while preserving systemic oxygenation better than atrial septostomy. Originally described in congenital heart disease, the Potts shunt has gained interest in PAH, particularly in children with idiopathic or heritable PAH. Outcomes have shown hemodynamic improvement and symptom relief in select patients; however, careful patient selection is crucial to avoid excessive systemic desaturation [81,85,86].

B. Pulmonary artery denervation (PADN)

Pulmonary artery denervation (PADN) is an emerging catheter-based intervention aimed at modifying sympathetic nerve activity in the pulmonary vasculature, thereby reducing pulmonary vascular resistance (PVR) and improving hemodynamics in PAH. PADN involves the radiofrequency ablation of sympathetic nerve fibers surrounding the main pulmonary artery. This procedure is performed via a catheter-based approach, typically accessing the pulmonary artery through the femoral vein. The PADN-1 trial was a first-in-human study that demonstrated significant reductions in mean pulmonary artery pressure (mPAP) and improvements in 6 min walk distance (6 MWD) at 3- and 6-month follow-ups [87]. The PADN-CFDA trial was a randomized–controlled trial comparing PADN plus standard PAH therapy to medical therapy alone, and it found a significant reduction in mPAP (−5.9 mmHg vs. −1.2 mmHg, *p* < 0.01) and improved cardiac output at 6 months [88]. A meta-analysis of PADN studies confirmed a consistent reduction in PVR and an improvement in WHO functional class in PAH patients undergoing PADN [89].

C. Lung Transplantation

Lung transplantation remains the definitive surgical intervention for patients with end-stage pulmonary arterial hypertension (PAH) who fail to respond to optimal medical therapy. Both bilateral lung transplantation (BLT) and heart–lung transplantation (HLT) are options, with BLT being preferred due to its lower rejection rates and better long-term survival compared to HLT.

**Indications and Outcomes**

Lung transplantation is indicated in PAH patients with refractory right heart failure, severe functional limitation (NYHA class III or IV), and poor prognosis despite maximal medical therapy. According to the registry of the International Society for Heart and Lung Transplantation (ISHLT), the median survival following lung transplantation in PAH patients is approximately 7 years, with improved survival in the era of modern immunosuppressive regimens [90]. An overall 5-year survival rate of 52% is seen following lung transplantation in patients with PAH, where early perioperative mortality remains a concern due to right ventricular failure. The bilateral lung transplant (BLT) is preferred as it has shown superior outcomes compared to single lung transplant (SLT) [91,92].

D. Balloon pulmonary angioplasty (BPA)

BPA is a catheter-based procedure aimed at treating chronic thromboembolic pulmonary hypertension (CTEPH) by dilating obstructed pulmonary arteries to improve blood flow. In clinical practice, BPA is primarily indicated for patients with CTEPH who are not suitable candidates for pulmonary endarterectomy (PE) or who have persistent or recurrent pulmonary hypertension post-surgery. Studies demonstrate significant improvements in hemodynamics, exercise capacity, and functional class after BPA, with reported 1-year survival rates of approximately 90% [93,94]. Contraindications include severe right heart failure, extensive disease that may not be amenable to intervention, or significant comorbidities that limit procedural success. Risks include vascular complications, pulmonary hemorrhage, and reperfusion injury, though these are generally outweighed by the benefits of improved symptoms and reduced pulmonary pressures. The benefit–risk profile is generally favorable when the treatment is performed by experienced teams, with long-term follow-up suggesting durable clinical improvements in most patients.

E. Pulmonary Thromboendarterectomy (PTE)

Pulmonary thromboendarterectomy (PTE), also referred to as pulmonary endarterectomy (PEA), is the definitive surgical treatment for chronic thromboembolic pulmonary hypertension (CTEPH). The procedure involves removing organized thromboembolic material from the pulmonary arteries to restore pulmonary vascular patency, reduce pulmonary vascular resistance, and improve right ventricular function. The perioperative mortality rate at expert centers is below 5%, with significant improvement in hemodynamics and symptoms postoperatively [95].

### 6.2. Decision-Making for Surgical Treatment [1,81,82]

Early referral for surgical evaluation improves outcomes, particularly in patients with rapidly deteriorating hemodynamics or refractory symptoms despite optimized medical therapy. Collaboration among pulmonologists, cardiologists, and thoracic surgeons ensures a comprehensive evaluation of risks and benefits. Advanced imaging and hemodynamic monitoring guide decision-making. Risk stratification tools such as the REVEAL risk calculator and ESC/ERS guidelines provide frameworks for quantifying surgical risks.

### 6.3. Emerging Surgical Strategies

#### 6.3.1. Hybrid Approaches

Hybrid procedures, combining atrial septostomy with medical therapies or mechanical support, are gaining traction for use in patients with severe hemodynamic compromise.

#### 6.3.2. Mechanical Support Devices

Right ventricular assist devices (RVADs) and extracorporeal membrane oxygenation (ECMO) are used as bridges to transplant or recovery. They provide hemodynamic stabilization in critically ill patients [81,96].

#### 6.3.3. Precision Medicine in Surgical PAH Management

Genetic profiling and molecular biomarkers are being integrated into risk assessment and post-surgical prognostication [97,98].

Surgical management in severe PAH is limited by high perioperative mortality, especially in patients with advanced right ventricular dysfunction. Future advancements should focus on refining patient selection criteria, improving surgical techniques, and incorporating novel perioperative care strategies.

Surgical strategies play a critical role in the management of severe PAH when medical therapies fail. Comprehensive risk assessment, multidisciplinary collaboration, and timely intervention are essential for optimizing outcomes. Emerging approaches, including hybrid procedures and mechanical support, hold promise for improving survival and quality of life in this high-risk population.

### 6.4. Palliative Care: An Overlooked Extra Panel of Support for PAH Therapy

Palliative care is a specialized mode of medical care designed to provide ease from symptoms and stress resulting from chronic illnesses, such as PAH and cancer. PAH undoubtedly affects physical, social and emotional functioning, contributing to the deterioration of the patient’s quality of life. Furthermore, the adverse effects of PAH-specific therapies add on to the worsening standard of living. Palliative care, being classified as an invasive and non-invasive therapy, overlaps with other treatment modalities (specific treatment and surgical intervention), as shown in Table 9.

Palliative care aids in improving functional status, hemodynamic parameters, and exercise tolerance, contributing to stabilizing the quality of life, and thus making it an essential part of the PAH treatment [81,99,100].

## 7. What Is on the Horizon?

### 7.1. Regenerative Medicine: A Potential Curative Approach for a Patient with PAH

As discussed, the management of PAH demands a holistic approach that encompasses both supportive and specific therapies. Current therapeutic modalities mainly revolve around the endothelin, NO–sGC–cGMP (nitric oxide–soluble Guanylate Cyclase–cyclic Guanosine Monophosphate), and prostacyclin pathways. Despite improved survival rates following recent therapies, introducing novel curative advanced therapy seems crucial. Modern therapeutic advancements (Regenerative Medicine) in PAH, comprising stem cell therapies, gene therapies, and epigenetic medicines, have shown promising results in preclinical studies, thus appearing as a potential cure with serious potential utility [101,102,103,104,105]. Regenerative Medicine mainly emphasizes repairing, replacing, or regenerating cells, tissues, or organs to restore their impaired function [102,103].

### 7.2. Stem Cell Therapy

**Figure 4:**
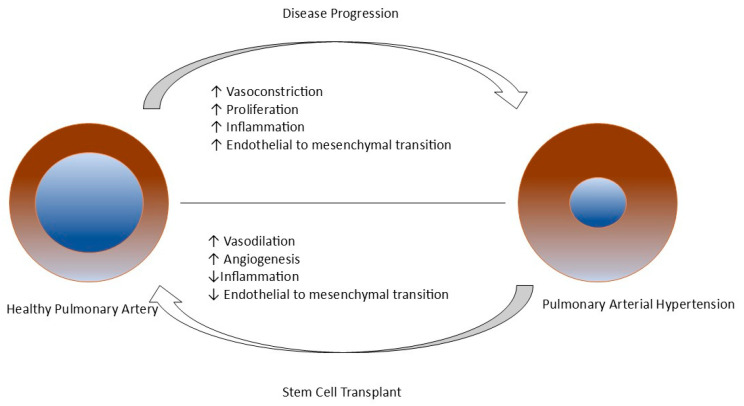
Mechanism of pulmonary arterial hypertension and role of stem cell transplants in its treatment [108,112].

Stem cells are undifferentiated cells that can self-renew and convert to specialized cells under exceptional circumstances. Depending on their differentiation potential, they can be classified as unipotent, oligopotent, multipotent, totipotent, or pluripotent stem cells [104,106,107,108]. They can be transplanted directly into the target site of the body or into the patient in the form of stem cells derived from mature cells following in vitro processing [109]. As these stem cells can differentiate into vascular cell lineages, they can potentially cure PAH [106,109,110]. Adult stem cell therapy is one of the trending topics in managing PAH due to its competency in restoring normal endothelial function and preventing the proliferation of smooth muscle cells in the pulmonary artery as shown in Figure 4. There are three significant varieties of stem cell therapy: endothelial progenitor cell therapy, mesenchymal stem cell therapy, and induced pluripotent stem cell therapy [105,108,111].

Endothelial progenitor cells (EPCs) are oligopotent stem cells predominantly found in bone marrow, with a tendency for endothelial cell differentiation. PAH is associated with a change in the number of circulating EPCs and the destruction of their migratory and adhesive capacity, leading to impaired vascular network formations [105,113]. EPC therapy is expected to improve PAH by differentiating into mature endothelial cells, repairing tissue, and restoring endothelial function. Furthermore, it can be diagnostically helpful by acting as circulating biomarkers to predict the risk of the disease [108,110,112,113]. During preclinical studies, EPC therapy improved hemodynamic parameters such as mean pulmonary arterial pressure (mPAP), right ventricular (RV) systolic pressure and pulmonary vascular resistance (PVR), and survival [114,115]. The PHACeT (Pulmonary Hypertension and Angiogenic Cell Therapy) trial demonstrated a short-term hemodynamic improvement and a long-term improvement in functional and quality of life assessments, with the two adverse effects being sepsis and death [116]. Recent clinical studies have also shown improvements in exercise capacity and hemodynamics [113,117]. These findings suggest EPC therapy has potential scope to be expanded in the future.

Mesenchymal stem cells (MSCs) are non-hematopoietic multipotent stem cells with the capacity to differentiate into various cell types, and an ability to release paracrine factors that contribute to the recovery of injured tissue. They can be easily isolated from bone marrow, adipose tissue, the umbilical cord, or the lungs [105,111,112,118]. MSC therapy is beneficial in patient with PAH, acting by repairing vascular function, inhibiting endothelial-to-mesenchymal transition, and inhibiting pro-inflammatory and pro-apoptotic factors in lung tissues. The principal mechanism by which it meets the treatment goal in PAH is related to paracrine effects [108,118,119]. Studies of MSC therapy in animal models have demonstrated the reversal of vascular remodeling, an increase in mPAP, pulmonary inflammation, and right ventricular hypertrophy [105,111,112,119]. The numerous advantages of this therapy make it an attractive alternative.

Induced pluripotent stem cells (iPSCs) are artificial pluripotent stem cells that are genetically reprogrammed from adult somatic cells to embryonic stem cell states using various transcription factors [105,108,110]. Huang et al. illustrated significant improvements in right ventricular systolic pressure and hypertrophy of the right ventricle in monocrotaline (MCT) rats [120]. As its limitations are apparent, including those related to tumorigenicity, genetic and epigenetic abnormalities, and long-term safety and efficacy, iPSCs are often used as cell models for investigating different phenotypes, drug screening, RNA sequencing, and the influence of gene mutation [104,108,110,121].

## 8. Gene Therapy

**Figure 5:**
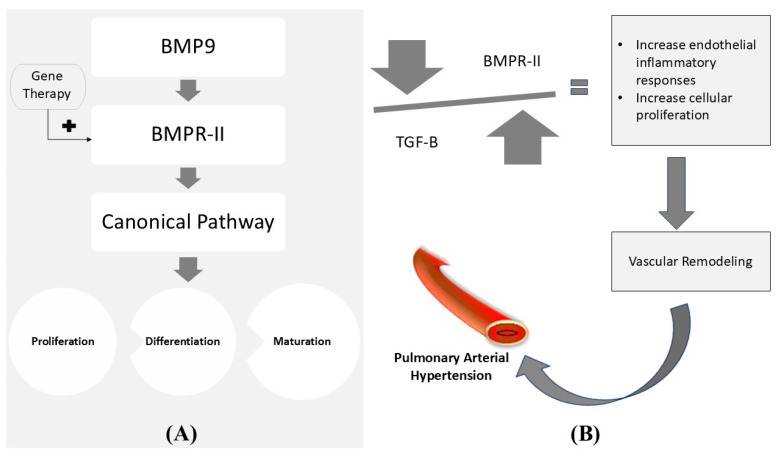
(**A**) The normal mechanism of stimulation of the canonical signaling pathway via the BMPR-II receptor, which ultimately controls the proliferation, differentiation, and maturation of the cell. (**B**) The imbalance in BMPR-II and TGF-β leading to PAH. The above diagram illustrates gene therapy’s possible utility in maintaining the normal canonical pathway. BMP9: Bone Morphogenic Protein-9. BMPR-II: Bone Morphogenic Protein Receptor Type II. TGF-β: Transforming Growth Factor-β [105,127,128].

The correction of mutated genes or site-specific modifications via genetics and bioengineering in order to achieve therapeutic goals is known as gene therapy [122]. Genetic factors play a significant role in PAH’s development, and primarily in Heritable PAH (HPAH). Among the numerous genetic mutations, BMPR-II (Bone Morphogenic Protein Receptor Type II), which is expressed predominantly in the pulmonary endothelium and undergoes complex interactions with the TGF-β (Transforming Growth Factor β) signaling pathway, is one of the more remarkable factors responsible for causing PAH [105,123,124,125]. BMPR-II gene therapy prevented increments in TGF-β and reduced right ventricular hypertrophy, pulmonary vascular resistance and remodeling [105,125,126]. These results reflect the applicability of gene therapy to the management of PAH as shown in Figure 5.

## 9. Epigenetic Medicines

The induction of heritable changes in the genome due to changes in gene expression without affecting the DNA sequence, such as DNA and RNA methylation, histone modifications, and non-coding RNA modification, is known as epigenetics. PAH-induced vascular cell changes occur at pathological and biochemical levels by affecting epigenetic-sensitive pathways. The advantage of epigenetic medicine, particularly DNA methylation, is that it is pharmacologically reversible, and allows for effective primary prevention [105,129,130,131].

Regenerative medicine is expected to induce reversible changes in PAH treatment, thus making it an attractive treatment strategy for exploration in the future.

## 10. Personalized and AI-Driven Treatment Approaches in PAH

The integration of precision medicine and artificial intelligence (AI) in pulmonary arterial hypertension (PAH) is revolutionizing patient care by enabling more tailored treatment strategies. AI-driven models utilize vast datasets, including clinical, genetic, and omics information, to predict individual responses to specific drugs, thus improving therapeutic outcomes and minimizing adverse effects [132]. Genetic testing plays a critical role in identifying mutations in genes like BMPR2, a key genetic marker linked to heritable PAH, which helps clinicians identify high-risk patients and customize treatments [133]. Additionally, biomarkers such as endothelin-1, BNP, and cytokines are increasingly used for patient stratification and in monitoring disease progression, helping to guide clinical decisions related to treatment intensity [134]. Advances in omics technologies—genomics, proteomics, and metabolomics—are shedding light on the molecular mechanisms underlying PAH. Genomics identifies genetic mutations or variants that predispose patients to PAH, while proteomics profiles the proteins involved in disease pathways, such as the endothelial nitric oxide synthase pathway [134,135]. Metabolomics examines the metabolic alterations associated with PAH, offering insights into disease mechanisms and potential therapeutic targets [136]. These technologies, combined with AI-driven prediction models, enable more precise treatment strategies that are tailored to the individual patient’s disease profile, offering hope for the better management of PAH in the future [132].

Lastly, there are many interesting trials due to commence soon. A few of them are listed in Table 10.

## 11. Conclusions

The initiation of a combination therapy early in the treatment plan is highly recommended for the management of this progressive and incurable disease. Long-term trials with morbidity and mortality benefits as outcome measures are needed to help us further understand the safety and effectiveness of treatment options.

Palliative care with different invasive and non-invasive measures could help promote patients’ comfort and alleviate distress. Though the management of pulmonary artery hypertension has achieved a significant profile with the advent of PAH-specific therapy, the quest for management options to cure or modify the disease course is still ongoing. Regenerative medicine, including stem cell therapy, gene therapy, and epigenetic therapy, represents a new horizon of PAH treatment that needs further exploration.

## 12. Materials and Methodology

### 12.1. Objective of the Review

This review aims to provide a comprehensive overview of the current understanding of pulmonary artery hypertension (PAH), including therapeutic strategies and emerging treatments.

### 12.2. Literature Search Strategy

A systematic literature search was conducted using electronic databases, including PubMed and Google Scholar, utilizing a combination of keywords such as “pulmonary hypertension”, “pulmonary arterial hypertension”, “endothelin receptor antagonists”, “phosphodiesterase-5 inhibitors”, “prostacyclin analogs”, “sotatercept”, “gene therapy”, “stem cell therapy”, etc. The search included studies published between 1990 and 2024.

### 12.3. Inclusion and Exclusion Criteria

Studies were included if they provided insights into current treatment strategies, their mechanism of action, surgical management strategies, or potential future options for the treatment of PAH. Both clinical trials and observational studies were considered. Different guidelines for pulmonary hypertension were included. References were tracked and included as appropriate. Non-English publications and studies unrelated to PH or its management were excluded.

## Figures and Tables

**Table 1:** Hemodynamic definitions of pulmonary arterial hypertension (PAH).

**Mean Pulmonary Artery Pressure (mPAP)**	≥20 mmHg
**Pulmonary Arterial Wedge Pressure (PAWP)**	≤15 mmHg
**Pulmonary Vascular Resistance (PVR)**	≥2 WU (Wood units)

**Table 2:** Classification of PAH.

Idiopathic PAH (IPAH) Heritable PAH (HPAH) Drug- and toxin-induced PAH (DT-PAH) PAH associated with: Connective tissue disease (CTD) HIV infection Portal hypertension Congenital heart disease (CHD) Schistosomiasis PAH long-term responders to calcium channel blockers PAH with overt features of venous/capillaries (PVOD/PCH) involvement. Persistent PH of the newborn syndrome.

**Table 3:** Relative risk (RR) of adverse effects of endothelin receptor antagonists (ERAs).

Drugs	Abnormal Liver Function	Peripheral Edema	Anemia
Bosentan	RR 2.93. (95% CI, 1.78–4.84)	RR 1.32. (95% CI, 0.87–2.00)	RR 1.39. (95% CI, 0.67–2.86)
Ambrisentan	RR 0.13. (95% CI, 0.01–1.13)	RR 1.62. (95% CI, 1.23–2.13)	RR 1.58. (95% CI, 0.88–2.82)
Macitentan	RR 0.78. (95% CI, 0.37–1.64)	RR 0.95. (95% CI, 0.68–1.31)	RR 3.42. (95% CI, 1.65–7.07)

**Table 4:** Comparison between PDE-5 inhibitors and sGC stimulators. CTEPH: chronic thromboembolic pulmonary hypertension.

Feature	PDE-5 Inhibitors (Sildenafil, Tadalafil)	sGC Stimulators (Riociguat)
Mechanism	Inhibits cGMP breakdown (NO-dependent)	Directly stimulates sGC (NO-independent)
Key indications	PAH (Group 1)	PAH (Group 1), CTEPH (group 4)
Dosing	Sildenafil (20 mg Three times daily), Tadalafil (40 mg daily)	Riociguat (0.5–2.5 mg Three times daily)
Combination allowed?	Not with sGC stimulators	Not with PDE-5 inhibitors
Nitrate contraindication?	Yes	Yes

**Table 5:** Primary endpoint (change in PVR from baseline to week 16).

	**M/T FDC**	**Macitentan**	**Treatment Effect**
**Reduction in PVR**	45%	23%	29% reduction
**Geometric mean ratio of change in PVR**	0.55 (95% CI = 0.50–0.60)	0.77 (95% CI = 0.69–0.87)	0.71 (95% CI = 0.61–0.82)
	**M/T FDC**	**Tadalafil**	**Treatment Effect**
**Reduction in PVR**	44%	22%	28% reduction
**Geometric mean ratio of change in PVR**	0.56 (95% CI = 0.52–0.60)	0.78 (95% CI = 0.72–0.84)	0.72 (95% CI = 0.64–0.80)

**Table 6:** Comparison of costs of different drugs used in PAH.

Medication	Formulation	Estimated Monthly Cost (USD)
Bosentan	Oral	10,000–12,000 [54]
Sildenafil	Oral	1000–2000 (brand); 200–400 (generic) [55]
Tadalafil	Oral	1300–2000 [55]
Epoprostenol	Intravenous	40,000–50,000 [56]
Treprostinil	Intravenous, subcutaneous, inhaled, oral	20,000–40,000 [57]
Selexipag	Oral	8000–10,000 [58]
Macitentan	Oral	11,000–13,000 [59]
Riociguat	Oral	8000–9000 [60]

**Table 7:** Summary of different classes of drugs currently in use for treatment of pulmonary arterial hypertension, with drug name, mechanism of action, and related clinical trials with their outcomes. cGMP, cyclic Guanosine Monophosphate; 6 MWD, 6 min walk distance; sGC, soluble Guanylate Cyclase (sGC); IP receptor, prostacyclin receptor; BMPR, bone morphogenetic protein receptor type 2.

Drug Class	Medications	Mechanism of Action	Some Related Clinical Trial(s) and Outcomes
Endothelin Receptor Antagonists (ERA)	Bosentan, Ambrisentan, Macitentan	Block endothelin-1 receptors (ET-A and/or ET-B), reducing vasoconstriction and proliferation	- **SERAPHIN (Macitentan):** Reduced morbidity and mortality [17]. - **AMBITION (Ambrisentan and Tadalafil combination):** Improved outcomes vs. monotherapy [52].
Phosphodiesterase-5 Inhibitors (PDE-5i)	Sildenafil, Tadalafil	Inhibit PDE-5, increasing cGMP and promoting vasodilation	- **SUPER-1 (Sildenafil):** Improved 6MWD [22]. - **PHIRST (Tadalafil):** Improved 6 MWD and reduced clinical worsening [24].
Soluble Guanylate Cyclase (sGC) Stimulators	Riociguat	Enhance sGC activity, increasing cGMP for vasodilation	- **PATENT-1 (Riociguat):** Improved 6 MWD and hemodynamics [29].
Prostacyclin Analogues	Epoprostenol, Treprostinil (IV/SQ/Inhaled/Oral), Iloprost	Prostacyclin receptor activation, promoting vasodilation and antiproliferation	- **REVEAL (Epoprostenol):** Improved survival [69]. - **FREEDOM-EV (Oral Treprostinil):** Reduced clinical worsening [70].
Prostacyclin Receptor Agonists	Selexipag	Selective IP receptor agonist, mimicking prostacyclin effects	- **GRIPHON (Selexipag):** Reduced morbidity/mortality [51].
Activin Signaling Inhibitor	Sotatercept	Restores BMPR2 signaling, promoting vascular remodeling	**STELLAR:** Improved 6MWD, PVR, and risk profile [67].
ERA + PDE-5i Combination pill	(Macitentan + Tadalafil)	Combines **Macitentan (ERA)** and **Tadalafil (PDE-5i)**	**A DUE:** Greater PVR and functional improvements vs. monotherapy [53].

**Table 8:** Management principles and treatment considerations in different subclasses of PAH. ASD, atrial septal defect; VSD, ventricular septal defect; PDA, patent ductus arteriosus; EIF2AK4, Eukaryotic translation initiation factor 2 alpha kinase 4.

Subgroup	General Management Principles	Treatment Consideration for PAH	Additional Considerations
Pulmonary Arterial Hypertension Associated with Connective Tissue Disease (PAH-CTD)	- Treat underlying autoimmune disease like Systemic Sclerosis according to the latest guideline [71] - Close coordination with multispecialty; example, rheumatology	- PDE5i, ERA, prostacyclin analogs - Same treatment algorithm as for patients with Idiopathic PAH	- Monitor closely for exacerbations and progression of underlying CTD
Pulmonary Arterial Hypertension Associated with Drugs and Toxins (DPAH)	- Immediately discontinue the agent causing PAH	- Same basic principles as the treatment for IPAH; PAH-directed therapy considered in intermediate- to high-risk group [1]	- Low-risk patients recommended to be re-evaluated at 3–4 months after discontinuation of offending agent; if hemodynamics does not normalize treat with PAH-specific medications [1]
HIV-Associated Pulmonary Arterial Hypertension (HIV-PH)	- Early diagnosis and initiation of antiretroviral therapy (ART) - Ensure optimal viral load control and immune function	- Current recommendations for PAH-specific medications are based on IPAH data - Initial monotherapy for PAH considered and, if needed, sequential combination therapy	- Monitor carefully for drug interactions
PAH Associated with Portal Hypertension	- Echocardiogram in patients with clinical features of PH and underlying liver disease and/or portal hypertension - Echocardiogram in patients being considered for Transjugular Portosystemic shunt or liver transplantation - Management of underlying liver disease and portal hypertension at a specialty center	- Initial monotherapy with PAH medication followed by sequential combination as necessary [72]	- Liver transplantation consideration if PVR is normal or near normal
PAH Associated with Adult CHD	In ASD, VSD, PDA with PVR M 3 WU, shunt closure recommended [1]	Limited data on the use of PAH-specific drugs; PDE5i and ERA might show improvements in functional class and hemodynamics in Eisenmenger [73]	Heart–lung transplantation is an option in patients who are not responsive to medical treatment; availability of organ and mortality in first year post-transplantation a concern.
PAH Associated with Schistosomiasis	Leading cause of PAH in Asia, Africa, South America	Uses of PAH-specific medications have improved survival per registry data [74]	
Pulmonary Veno-Occlusive Disease (PVOD)	Combination of clinical, radiological, blood gas, pulmonary function test and genetic testing (EIF2AK4 mutation) recommended in PAH with suspicion of venous/capillary involvement	Risk of pulmonary edema with the use of pulmonary vasodilators	Referral to a transplant center for evaluation is recommended

**Table 9:** Palliative care in end-stage PAH patients. AS: atrial septostomy. ECMO: extracorporeal membrane oxygenation. RVAD: right ventricle assist device [81,99].

**Invasive therapy**	Right to left shunting (AS, Potts shunt) Pulmonary artery denervation Others: RVAD, right ventricular pacing, ECMO
**Non-invasive therapy**	Pain management Symptomatic treatment Treatment of underlying psychiatric disorders Specific therapy
**Others**	Counseling Financial assistance

**Table 10:** Some of the upcoming trials in PAH.

Title	Primary Outcome Measures	Time Frame
Positioning Imatinib for Pulmonary Arterial Hypertension (PIPAH)	Identifying the highest tolerated dose Change in pulmonary vascular resistance (PVR)	12 months 24 months
Clinical Trial of 2-hydroxbenzylamine (2-HOBA) in Pulmonary Arterial Hypertension	Changes in acetylated Superoxide Dismutase 2 (SOD2) and Long-chain acyl-CoA dehydrogenase (LCAD) in plasma.	Baseline and 12-weeks
Apabetalone for Pulmonary Arterial Hypertension (APPROACH-2)	Placebo-corrected change from baseline in PVR at week 24	Baseline and 24 weeks
Metabolic Remodeling in Pulmonary Arterial Hypertension (PAH)	Change in ratio of oxidative metabolism to glycolysis	Baseline and 6 months
